# Intrapersonal Stability of Plasma Metabolomic Profiles over 10 Years among Women

**DOI:** 10.3390/metabo12050372

**Published:** 2022-04-20

**Authors:** Oana A. Zeleznik, Clemens Wittenbecher, Amy Deik, Sarah Jeanfavre, Julian Avila-Pacheco, Bernard Rosner, Kathryn M. Rexrode, Clary B. Clish, Frank B. Hu, A. Heather Eliassen

**Affiliations:** 1 Channing Division of Network Medicine, Brigham and Women’s Hospital and Harvard Medical School, Boston, MA 02115, USA; stbar@channing.harvard.edu (B.R.); nhahe@channing.harvard.edu (A.H.E.); 2 Department of Nutrition, Harvard T.H. Chan School of Public Health, Boston, MA 02115, USA; cwittenbecher@hsph.harvard.edu (C.W.); nhbfh@channing.harvard.edu (F.B.H.); 3 German Institute of Human Nutrition Potsdam-Rehbruecke, 14558 Nuthetal, Germany; 4 Broad Institute of MIT and Harvard, Cambridge, MA 02142, USA; adeik@broadinstitute.org (A.D.); sjean@broadinstitute.org (S.J.); jravilap@broadinstitute.org (J.A.-P.); clary@broadinstitute.org (C.B.C.); 5 Division of Women’s Health, Brigham and Women’s Hospital and Harvard Medical School, Boston, MA 02115, USA; krexrode@bwh.harvard.edu; 6 Department of Epidemiology, Harvard T.H. Chan School of Public Health, Boston, MA 02115, USA

**Keywords:** lipids and lipid-related metabolites, polar metabolites, within-person stability, unknown metabolite features

## Abstract

In epidemiological studies, samples are often collected long before disease onset or outcome assessment. Understanding the long-term stability of biomarkers measured in these samples is crucial. We estimated within-person stability over 10 years of metabolites and metabolite features (*n* = 5938) in the Nurses’ Health Study (NHS): the primary dataset included 1880 women with 1184 repeated samples donated 10 years apart while the secondary dataset included 1456 women with 488 repeated samples donated 10 years apart. We quantified plasma metabolomics using two liquid chromatography mass spectrometry platforms (lipids and polar metabolites) at the Broad Institute (Cambridge, MA, USA). Intra-class correlations (ICC) were used to estimate long-term (10 years) within-person stability of metabolites and were calculated as the proportion of the total variability (within-person + between-person) attributable to between-person variability. Within-person variability was estimated among participants who donated two blood samples approximately 10 years apart while between-person variability was estimated among all participants. In the primary dataset, the median ICC was 0.43 (1st quartile (Q1): 0.36; 3rd quartile (Q3): 0.50) among known metabolites and 0.41 (Q1: 0.34; Q3: 0.48) among unknown metabolite features. The three most stable metabolites were N6,N6-dimethyllysine (ICC = 0.82), dimethylguanidino valerate (ICC = 0.72), and N-acetylornithine (ICC = 0.72). The three least stable metabolites were palmitoylethanolamide (ICC = 0.05), ectoine (ICC = 0.09), and trimethylamine-N-oxide (ICC = 0.16). Results in the secondary dataset were similar (Spearman correlation = 0.87) to corresponding results in the primary dataset. Within-person stability over 10 years is reasonable for lipid, lipid-related, and polar metabolites, and varies by metabolite class. Additional studies are required to estimate within-person stability over 10 years of other metabolites groups.

## 1. Introduction

In epidemiological studies, samples are often collected long before disease onset or outcome assessment. Within the Nurses’ Health Studies (NHS) and NHSII, several nested case–control studies investigated prospective associations of plasma biomarkers measured in samples collected >10 years before disease onset with risk of developing cancer and other chronic diseases. For example, total circulating carotenoids measured up to 20 years before diagnosis were associated with decreased risk of developing breast cancer [1]. In contrast, high plasma prolactin levels measured ≥10 years before diagnosis were not associated with increased risk of breast cancer while measures <10 years before diagnosis were associated with increased risk [2], emphasizing that understanding how the timing of biomarkers is related to risk is critical to elucidating disease etiology. Thus, understanding the long-term within-person stability of biomarkers measured in these samples is crucial.

The metabolome, which reflects the integrated effects of the genetic background, lifestyle, and environmental factors [3], is of particular interest in epidemiologic studies. Metabolites measured up to 23 years before diagnosis were associated with risk of ovarian cancer in NHS and NHSII [4,5] reflecting long-term disease–biomarker associations. Multiple studies reported on the stability of metabolomics profiles with respect to preanalytical conditions, storage time, and repeated freeze–thaw cycles [6,7,8,9,10,11,12,13,14,15,16,17,18,19,20,21], and participant characteristics such as gender, age, fasting status, and body mass index (BMI) [22,23,24,25,26,27,28,29,30,31,32]. We and others reported previously on the short-term within-person stability or metabolites [33,34,35]. However, data on the long-term within-person stability of metabolomics are lacking. Here, we assessed metabolomics within-person stability over 10 years in two separate prospective case–control studies nested within a large epidemiological study, the NHS.

## 2. Results

### 2.1. Study Population Characteristics

Women in the primary dataset had a mean age of 56 years at the first blood collection (*n* = 1880) and 66 years at the second blood collection (*n* = 1184). Participant characteristics were similar at the two blood collections with some exceptions: more women reported being postmenopausal, past smokers, and fasting >8 h at the second blood collection compared to the first blood collection. Women in the secondary dataset (first collection *n* = 1456, second collection *n* = 488) were similar to the fasting women (first collection *n* = 1309, second collection *n* = 1062) in the primary dataset.

### 2.2. Metabolite Profile Stability over 10 Years in the Primary Dataset

Metabolites had a median intra-class correlation (ICC) of 0.43 (1st quartile (Q1): 0.36; 3rd quartile (Q3): 0.50; Table 1). ICCs among lipids and lipid-related metabolites were similar to ICCs among polar metabolites (median ICC = 0.44 vs.0.42; Wilcoxon test *p* = 0.06). ICCs differed significantly comparing metabolites with coefficient of variation (CV) among quality control samples < 25% to metabolites with CV ≥ 25% (median ICC: 0.44 vs. 0.34; Wilcoxon test *p* < 0.01). The median % difference in metabolite levels between the two collections, calculated from raw values, was −5.25% (Q1: −14.80%, Q3: 0.92%; Supplementary Table S1). ICCs among known metabolites were slightly different from ICCs among unknown metabolite features (median ICC: 0.43 vs. 041; Wilcoxon test *p* < 0.01) but showed similar patters across metabolite subsets (Supplementary Material; Supplementary Table S2). The three most stable metabolites were N6,N6-dimethyllysine (ICC = 0.82), dimethylguanidino valerate (ICC = 0.72) and N-acetylornithine (ICC = 0.72) while the three least stable metabolites were palmitoylethanolamide (ICC = 0.05), ectoine (ICC = 0.09), and trimethylamine-N-oxide (ICC = 0.16). Results for all known metabolites, including CVs from blinded quality control samples, between- and within-person CV, mean % difference, and ICCs across participant strata are included in Supplementary Table S1.

**Figure 1:**
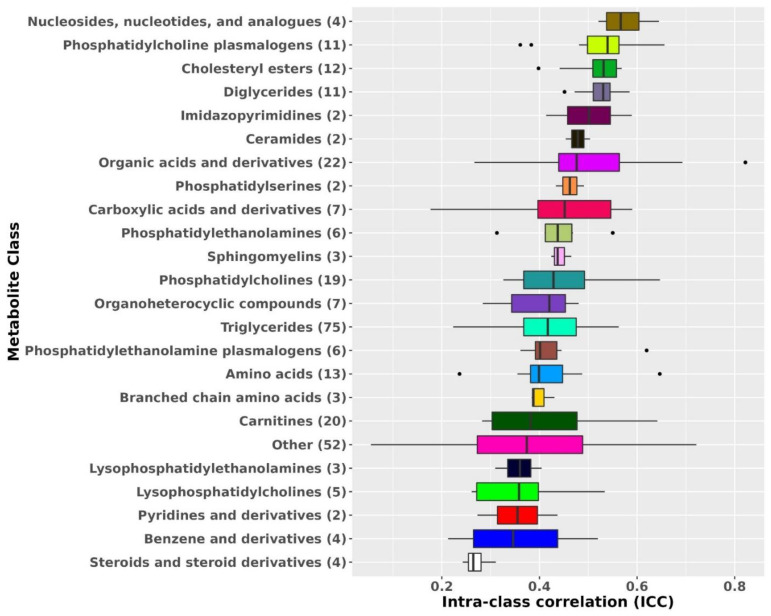
Metabolomic profiles stability over 10 years by metabolite class. Results from known metabolites are included in this figure. Metabolite classes with less than two metabolites were added to the class Other. ICCs beyond the whiskers (outliers) are plotted individually as black dots. The left whisker extends from the left hinge of the box (25th percentile) to the smallest value but no further than 1.5*IQR (inter-quartile range). The right whisker extends from the right hinge of the box (75th percentile) to the largest value but no further than 1.5*IQR (inter-quartile range).

Metabolite ICCs also varied by metabolite class (Figure 1). The most stable metabolite classes were nucleosides, nucleotides, and analogues (median ICC: 0.57), phosphatidylcholine (PC) plasmalogens (median ICC: 0.54), diglycerides (DG; median ICC: 0.53), and cholesteryl esters (CE; median ICC: 0.53). The least stable metabolite classes were steroids and steroid derivatives (median ICC: 0.26), benzene and derivatives (median ICC: 0.35), and pyridines and derivatives (median ICC = 0.36).

We observed statistically significant, albeit small, differences in metabolite ICCs across participant strata in sensitivity analyses (Table 2, Supplementary Table S1, and Supplementary Figure S1). Metabolite ICCs estimated among all women (median ICC = 0.43) were slightly different from ICCs estimated among fasting women (median ICC = 0.45; paired Wilcoxon test *p* < 0.01), from ICCs estimated among women with stable BMI (median ICC = 0.43; paired Wilcoxon test *p* < 0.01), from ICCs estimated among women with a change in BMI (median ICC = 0.41; paired Wilcoxon test *p* < 0.01), from ICCs estimated among postmenopausal women not using hormone therapy at either collection (median ICC = 0.44; paired Wilcoxon test *p* < 0.01), and from ICCs estimated among control women (median ICC = 0.44; paired Wilcoxon test *p* < 0.01). While statistically significant, the magnitude of impact on ICCs by these characteristics is fairly small. Similar patters were observed among unknown metabolite features (Supplementary material, Supplementary Table S3).

Notably, metabolites stable among all women were also stable when assessed in different participant strata (for example, N6,N6-dimethyllysine ICC ranges between 0.82 and 0.84 across participant strata; Table 3). Similarly, metabolites with low ICC among all women were also not stable when assessed in the different participant strata (for example, palmitoylethanolamide ICC ranges between 0.03 and 0.06 across subgroups; Table 3).

### 2.3. Metabolite Profile Stability over 10 Years in the Secondary Dataset

**Figure 2:**
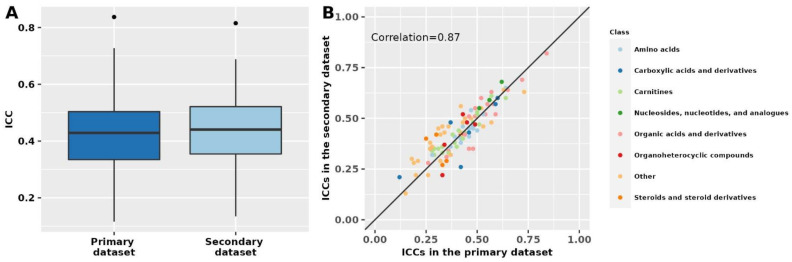
Metabolomics stability over 10 years in the primary and secondary datasets. The primary dataset was restricted to polar metabolites and fasting women to match the secondary dataset. Intra-class correlations (ICCs) in the two datasets are shown as boxplots (panel **A**) and by metabolite class in a scatter plot (panel **B**). The correlation was estimated using Spearman’s rank correlation coefficient. In panel (**A**), ICCs beyond the whiskers (outliers) are plotted individually as black dots. The lower whisker extends from the lower hinge of the box (25th percentile) to the smallest value but no further than 1.5*IQR (inter-quartile range). The upper whisker extends from the upper hinge of the box (75th percentile) to the largest value but no further than 1.5*IQR (inter-quartile range).

Results in the secondary dataset, which included known polar metabolites and fasting women, were similar to results for known polar metabolites among fasting women in the primary dataset (Spearman correlation = 0.87; Figure 2, Supplementary Table S4). In the secondary dataset, the median ICC among known metabolites was 0.43 (Q1: 0.34; Q3: 0.51), similar to the median ICC of 0.43 (Q1: 0.35; Q3: 0.52) among unknown metabolite features. Among known metabolites for which we were able to estimate ICCs in both datasets (*n* = 105), 52 (50%) metabolites had % ICC absolute difference <10% and 82 (78%) had % absolute difference <20%. The metabolites with the lowest % absolute ICC difference between the two datasets were 1,7-dimethyluric acid (primary dataset ICC: 0.42; secondary dataset ICC: 0.42), caffeine (primary dataset ICC: 0.42; secondary dataset ICC: 0.43), and creatinine (primary dataset ICC: 0.60; secondary dataset ICC: 0.60). Similarly, 17 metabolites ranked among the top 25 most stable metabolites in the primary dataset were also ranked among the 25 most stable metabolites in the secondary dataset.

## 3. Discussion

We estimated within-person stability over 10 years for 5938 metabolites (295 known compounds and 5643 unknown metabolite features) among 1880 women. Most metabolites were reasonably stable over 10 years with a median ICC of 0.43 for known metabolites and 0.41 from unknown metabolite features. Within-person stability over 10 years varied by metabolite class. In secondary analyses, we performed a partial replication of polar metabolites in another data set of 1456 fasting women; findings were similar.

To the best of our knowledge, this is the first study to assess within-person stability of metabolomic profiles over 10 years. Within-person stability over 10 years was attenuated when compared to within-person stability over 1–2 years [33,34,35]. While 61% of metabolites showed ICCs > 0.5 over 1 year, only 25% of the metabolites in our study had similar ICCs over 10 years [35]. However, the metabolite classes that were the most stable over 1–2 years were also the most stable over 10 years [33]. For example, cholesteryl esters, phosphatidylcholine plasmalogens, and diglycerides had high median ICCs over 1–2 years (median ICC: 0.73–0.76) and over 10 years (median ICC: 0.53–0.55). Nucleosides, nucleotides, and analogues were the most stable class over 10 years (10 years ICC = 0.57, 1–2 years ICC = 0.55). Steroid and steroid derivatives was the least stable class over 1–2 years (median ICC = 0.36) and over 10 years (median ICC = 0.26) [33]. Although metabolite levels are associated with personal characteristics [22,23,24,25,26,27,28,29,30,31,32], these characteristics have significant but small effects on both short-term (age, gender, fasting [35]) and long-term within-person stability (fasting, BMI, postmenopausal hormone therapy use) with effects varying by metabolite class [21,34].

Our results show that although the within-person stability decreases over time, metabolites are reasonably stable over 10 years. Homeostasis, the body’s ability to maintain fairly steady conditions, may be one of the underlying factors driving stability in some metabolites. The reduced long-term stability over 10 years compared to 1–2 years reflects greater within-person variation over longer periods of time. True changes in metabolite levels over long periods of time represent the most important source of variability. Changes over 10 years in personal, behavioral, and lifestyle factors such as age, BMI, menopausal status, exposure to postmenopausal hormone therapy, diet, and physical activity are likely to affect metabolite levels. For example, acetaminophen, a drug often used sporadically for different types of aches and pains, had a low within-person stability, likely reflecting different windows of exposure. Furthermore, some diet-related metabolites (e.g., trimethylamine-N-oxide (TMO) [36], pipecolic acid [37]) tended to show low within-person stability over 10 years. Notably, within person stability over time among triglycerides (TAG) varied by saturation level and length of the fatty acyl chains. Most highly unsaturated TAGs with long fatty acyl chains, which are associated with long-term vegetable intake [37], tended to be more stable over time compared to less unsaturated TAGs with shorter fatty acyl chains. While changes in behaviors and exposures result in a reduced 10 years within-person stability of metabolites, it is important to note that we expect changes in metabolite levels in response to changes in these factors. Furthermore, we study metabolites to identify new risk and disease biomarkers because they reflect changes in these factors and are considered a representation of the metabolic state of an individual, the integrated effects of their genetic background, lifestyle, and environmental exposure [3]. A critical feature of some of the most widely applied clinical disease risk markers, such as standard blood lipids, is their responsiveness to pharmacological and lifestyle-based risk prevention. Long-term storage may represent another source of variation. However, the samples in these cohorts are stored at ultra-low temperatures (≤−130 °C) which were shown to limit the negative effects of long-term storage [38]. Additionally, we have identified metabolites measured in these long-term stored samples that were significantly associated with cancer and other chronic diseases in multiple studies (e.g., pancreatic [39], ovarian [4,5], breast [40,41] and prostate cancer [42], rheumatoid arthritis [43], and cardiovascular disease [44]), suggesting that storage time does not substantially impact biomarker–disease associations. Furthermore, for a considerable subset of metabolites (e.g., cotinine, trigonelline, caffeine, pantothenate, C45:3 TAG, C54:9 TAG), we also observed relatively large median % differences over 10 years (−47%/+58%) and, at the same time, reasonably high ICCs (>0.4), suggesting that potential changes in metabolite levels due to long term-storage are similar across individuals.

For a subset of metabolites, two additional sources of variation must be considered. To leverage the large coverage of the assay, we did not exclude metabolites with low technical reproducibility from the analysis. Furthermore, the samples in this analysis are subject to delayed processing (24–48 h after sample collection). We excluded metabolites where we have documented variation with a delay in processing [33], but this information was not available for all known metabolites, and not available for any of the unknown metabolite features. Notably, both factors would result in a potential underestimation of long-term within-person stability.

The 10-year within-person stability of metabolites is similar to other plasma biomarkers measured in the NHS. For example, postmenopausal hormone levels (estradiol ICC = 0.69, testosterone ICC = 0.71, sex hormone-binding globulin ICC = 0.74, and dehydroepiandrosterone sulfate ICC = 0.54) [45], 25-hydroxyvitamin D (ICC = 0.51) [46], and prolactin (ICC = 0.39) [2] all showed high or moderate within-person reproducibility over 10 years, whereas dietary biomarkers such as carotenoids (ICCs ranged between 0.3 for β-carotene and 0.54 for lutein and zeaxanthin) [1] and fluorescent oxidation products (ICCs range from 0.14 to 0.30) [47] showed moderate or modest long-term within-person stability. While long-term stability should be factored into result interpretation, many of the most predictive and widely used biomarkers have similar within-person stability over 10 years in this cohort. For example, plasma cholesterol has a 10-year ICC of 0.39 and is highly predictive of coronary artery disease risk in our [48,49,50] and other cohorts.

Our study has several strengths and limitations. We were able to assess within-person stability over 10 years for over 295 known metabolites (polar metabolites and lipids and lipid-related metabolites) and 5643 unknown metabolite features. However, our study did not include other metabolite groups such as fatty acids, carbohydrates, alcohols, and vitamins. We had a large sample size and were able to conduct a partial replication for a subset of the measured metabolites among fasting women, but our study included mostly Caucasian women limiting its generalizability. It should also be noted that the samples used in this analysis were subject to delayed processing (blood collection characteristic in the NHS). While we excluded metabolites known to vary with a delay in processing, this information was not available for all analyzed metabolites. Additionally, we did not exclude metabolites with low technical reproducibility. Due to these factors, the ICCs presented here may include variation due to technical reproducibility and/or differential delay in processing between the two blood collections which can potentially result in an underestimation of within-person stability over time.

In summary, our study showed that metabolites are reasonably stable over 10 years, a time interval characteristic of prospective epidemiologic studies of chronic disease. In a secondary dataset, we were able to replicate our findings for a subset of metabolites among fasting women. Stability over 10 years varied by metabolite class. While the 10-year stability of metabolites should be an important factor when interpreting results, it is equally important to consider the sources of variation that influence long-term within-person stability of metabolites. Findings from this study represent a comprehensive resource for the design of future studies into disease risk associations of specific metabolites and/or metabolite classes.

## 4. Materials and Methods

### 4.1. Study Population

**Figure 3:**
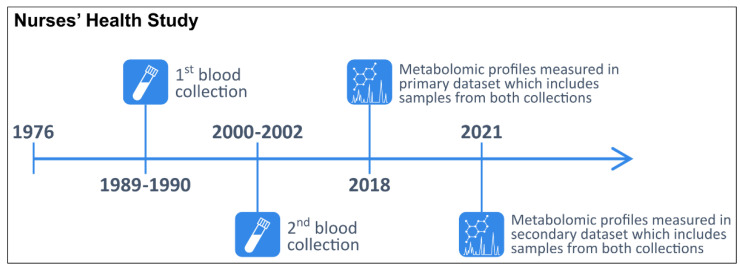
Timeline of the two blood collections and metabolomic profiling in the primary and secondary datasets within the Nurses’ Health Study.

In 1976, 121,701 female registered nurses aged 30–55 years enrolled in the NHS with the return of a mailed questionnaire [51]. Participants have been followed biennially with questionnaires collecting information on reproductive history, lifestyle factors, diet, medication use, and new disease diagnoses. In 1989–1990, 32,826 NHS participants aged 43–69 years contributed blood samples, as previously described [52]. In 2000–2002, 18,473 of these women aged 53–80 years donated a second sample using a similar protocol. The study design and timeline are summarized in Figure 3.

The primary dataset was obtained from a prospective breast cancer case–control study nested within the NHS (Table 4) [40,41]. Incident cases of breast cancer (*n* = 940) were identified after the second blood collection among women who had no reported cancer (other than non-melanoma skin). In total, 1880 women donated a sample during the first blood collection and 1184 women donated a second blood sample approximately 10 years later. Samples from the first collection were stored for approximately 28 years while samples from the second collection were stored for approximately 18 years before metabolomic profiling.

The secondary dataset is from a prospective diabetes case–control study nested within the NHS (Supplementary Table S5) [53]. Incident cases of diabetes (*n* = 728) were identified after the second blood collection. In total, 1456 women donated samples during the first blood collection and 488 women donated a second blood sample approximately 10 years later. Samples from the first collection were stored for approximately 31 years while samples from the second collection were stored for approximately 21 years before metabolomic profiling.

The study protocol was approved by the institutional review boards of the Brigham and Women’s Hospital and Harvard T.H. Chan School of Public Health, and those of participating registries as required. The return of the self-administered questionnaire and blood sample was considered to imply consent.

### 4.2. Blood Collection Methods

The same protocol was used for both blood collections. Briefly, participants had their blood drawn in sodium heparin tubes at a nearby clinic or by their colleagues, and the blood samples were shipped with an ice pack via overnight courier to our laboratory. Whole blood samples were centrifuged (2500 revolutions per minute (RPM) for 20 min at 4 °C) and aliquoted into 5 mL plasma, red blood cells and white blood cells cryotubes. Plasma samples were stored in the vapor phase of liquid nitrogen (LN2) freezers (temperature ≤ −130 °C; alarmed and monitored 24 h a day) with LN2-rated gasketed screw tops since collection. At the time of blood collection, participants also completed a questionnaire regarding time since last meal and time of day when they completed blood collection.

### 4.3. Metabolite Profiling

Metabolic profiles were assayed through a metabolomic profiling platform at the Broad Institute using a liquid chromatography tandem mass spectrometry (LC-MS) method designed to measure polar metabolites such as amino acids and lipids [54,55,56]. The relative abundance of each metabolite was determined by the integration of LC-MS peak areas, which are proportional to metabolite concentrations. For each measurement method (polar metabolites and lipids), pooled plasma reference samples were included every 20 samples and results were standardized using the ratio of the value of the sample to the value of the nearest pooled reference multiplied by the median of all reference values for the metabolite. In each dataset, samples collected at both collections from the same individual and matched case–control pairs were run adjacent to each other. Therefore, variability in platform performance across samples within individuals was limited. In addition, 426 quality control (QC) samples, to which the laboratory was blinded, were also profiled; coefficients of variation (CV) were calculated from these samples and presented in Supplemental Table S1. QC samples were randomly distributed among the participants’ samples.

Hydrophilic interaction liquid chromatography (HILIC) analyses of water-soluble metabolites in the positive ionization mode were conducted using an LC-MS system composed of a Shimadzu Nexera X2 U-HPLC (Shimadzu Corp.; Marlborough, MA, USA) coupled to a Q Exactive mass spectrometer (Thermo Fisher Scientific; Waltham, MA, USA). Metabolites were extracted from plasma (10 µL) using 90 µL of acetonitrile/methanol/formic acid (74.9:24.9:0.2 *v*/*v*/*v*) containing stable isotope-labeled internal standards (valine-d8, Sigma-Aldrich; St. Louis, MO; and phenylalanine-d8, Cambridge Isotope Laboratories; Andover, MA, USA). The samples were centrifuged (10 min, 9000× *g*, 4 °C), and the supernatants were injected directly onto a 150 × 2 mm, 3 µm Atlantis HILIC column (Waters; Milford, MA, USA). The column was eluted isocratically at a flow rate of 250 µL/min with 5% mobile phase A (10 mM ammonium formate and 0.1% formic acid in water) for 0.5 min followed by a linear gradient to 40% mobile phase B (acetonitrile with 0.1% formic acid) over 10 min. MS analyses were carried out using electrospray ionization in the positive ion mode using full scan analysis over 70–800 m/z at 70,000 resolution and 3 Hz data acquisition rate. Other MS settings were: sheath gas 40, sweep gas 2, spray voltage 3.5 kV, capillary temperature 350 °C, S-lens RF 40, heater temperature 300 °C, microscans 1, automatic gain control target 1 × 10^6^, and maximum ion time 250 ms. Metabolites measured with this method will be referred to as HILIC-positive metabolites.

Plasma lipids were profiled using a Shimadzu Nexera X2 U-HPLC (Shimadzu Corp.; Marlborough, MA, USA). Lipids were extracted from plasma (10 µL) using 190 µL of isopropanol containing 1,2-didodecanoyl-sn-glycero-3-phosphocholine (Avanti Polar Lipids; Alabaster, AL, USA). After centrifugation, supernatants were injected directly onto a 100 × 2.1 mm, 1.7 µm ACQUITY BEH C8 column (Waters; Milford, MA, USA). The column was eluted isocratically with 80% mobile phase A (95:5:0.1 *v*/*v*/*v* 10 mM ammonium acetate/methanol/formic acid) for 1 min followed by a linear gradient to 80% mobile-phase B (99.9:0.1 *v*/*v* methanol/formic acid) over 2 min, a linear gradient to 100% mobile phase B over 7 min, then 3 min at 100% mobile-phase B. MS analyses were carried out using electrospray ionization in the positive ion mode using full scan analysis over 200–1100 m/z at 70,000 resolution and 3 Hz data acquisition rate. Other MS settings were: sheath gas 50, in source CID 5 eV, sweep gas 5, spray voltage 3 kV, capillary temperature 300 °C, S-lens RF 60, heater temperature 300 °C, microscans 1, automatic gain control target 1 × 10^6^, and maximum ion time 100 ms. Lipid identities were denoted by total acyl carbon number and total double bond number. Metabolites measured with this method will be referred to as C8-positive metabolites.

Raw data from orbitrap mass spectrometers were processed using TraceFinder 3.3 software (Thermo Fisher Scientific; Waltham, MA, USA) and Progenesis QI (Nonlinear Dynamics; Newcastle upon Tyne, UK). For analytical quality control, pooled plasma reference samples and mixtures of synthetic metabolite reference standards were analyzed at the beginning and end of sample queues to assure stable analytical performance, internal standard signals were evaluated in each sample to ensure consistent sample volume injections, and pooled plasma QC samples were inserted into the analytical queue at a frequency of 5% to evaluate analytical repeatability of each metabolite. Plasma samples were thawed on ice prior to aliquoting. As the aliquots for the LC-MS methods were prepared from each sample, a pooled plasma sample was created by placing an additional 10 μL aliquot from each sample into a 50 mL conical centrifuge tube. The pooled plasma sample was maintained on dry ice while samples were being aliquoted to promote rapid freezing and stored at −80 °C in between sample batches until all additions were made. The pooled plasma was then thawed on ice, mixed by vortexing, and sub-aliquoted to create pooled plasma QC samples for each LC-MS method. For each method, metabolite identities were confirmed using mixtures of authentic reference standards (that were previously individually identified in human plasma based on matching retention times, m/z, and MS/MS spectra) or reference samples (see Supplementary Table S6).

After exclusion of metabolites not stable with a delay in processing which is characteristic of the two blood collections [33] (*n* = 35) and those missing in >10% of the participants who donated two samples (20 known compounds and 1069 unknown metabolite features), the primary dataset included 5938 metabolites (295 known compounds and 5643 unknown metabolite features) measured at both blood collections. In total, 2519 lipids and lipid-related metabolites (153 known compounds and 2366 unknown metabolite features) were measured with the C8 column in positive mode while 3419 polar metabolites (142 known compounds and 3277 unknown metabolite features) were measured with the HILIC column in positive mode. Most of the known metabolites (*n* = 253; 86%) and 47% (*n* = 2655) of the unknown metabolite features had coefficients of variation (CV) <25%. All metabolites were included in this study.

Similarly, after exclusion of metabolites not stable with a delay in processing which is characteristic of the two blood collections [33] (*n* = 34) and those missing in >10% of the participants who donated two samples (16 known compounds and 427 unknown metabolite features), the secondary dataset included 3209 polar metabolites (202 known compounds and 3007 unknown metabolite features) measured at both blood collections. The secondary dataset did not include lipids and lipid-related metabolites. Most of the known metabolites (*n* = 171; 85%) and 44% (*n* = 1326) of the unknown metabolite features had CV < 25%. All metabolites were included in this study.

### 4.4. Statistical Analysis

Metabolite values were transformed to probit scores within each blood collection and dataset. We estimated within-person stability over 10 years by calculating intra-class correlation (ICC) using liner mixed models with participant IDs as a random effect. We followed the approach developed by Dr. Rosner et al. [57] to estimate ICCs on the probit scale and transform these back to the original scale. Within-person variability was estimated among participants who donated two blood samples approximately 10 years apart (*n* = 1184) while between-person variability was estimated among all participants (*n* = 1880). ICCs were calculated as the proportion of the total variability (within-person + between-person) attributable to between-person variability. ICCs range between 0 and 1, with 0 indicating no stability over time (no between-person variability; all variability is attributable to within-person variability) and 1 indicating perfect stability over time (no within-person variability; all variability is attributable to between-person variability). ICCs close to 1 reflect a higher proportion of the total variability due to between-person variability. We also calculated median % change in metabolite levels over 10 years on the original scale. In sensitivity analyses, we restricted to fasting women (*n* = 1309 of which 765 donated 2 samples), women with stable BMI (≤2 kg/m^2^ change in BMI between the two blood collections, *n* = 706), women with change in BMI (>2 kg/m^2^ change in BMI between the two blood collections, *n* = 478), postmenopausal women not using postmenopausal hormone therapy at either time point (*n* = 577 of which 223 donated 2 samples), and women without breast cancer at both blood collections (*n* = 940 of which 592 donated 2 samples). We compared ICCs between metabolite groups (e.g., ICCs for lipids vs. ICCs for polar metabolites) using the Wilcoxon rank sum test and between participant groups (e.g., ICCs estimated among all women vs. ICCs estimated among fasting women) using the Wilcoxon signed rank test for paired observations. The secondary dataset included only women who were fasting for >8 h. To assess differences in ICCs between the two datasets, we estimated the Spearman correlation and calculated % absolute change in ICCs between the primary and secondary dataset.

## Figures and Tables

**Table 1:** Intra-class correlations (ICC) among 295 metabolites, by metabolite subsets, in the primary dataset.

Metabolites	Median	Quartile 1−Quartile 3
All metabolites	0.43	0.36–0.50
Lipids and lipid-related metabolites	0.44	0.38–0.51
Polar metabolites	0.42	0.33–0.49
Metabolites with CV < 25%	0.44	0.38–0.51
Metabolites with CV ≥ 25%	0.34	0.28–0.42

CV: coefficient of variation from blinded quality control samples.

**Table 2:** Metabolomic profiles stability over 10 years for known metabolites (*n* = 295). ICCs were estimated among all women (*n* = 1880 of which 1184 donated 2 samples), fasting women (*n* = 1309 of which 765 donated 2 samples), among women with stable BMI (*n* = 706) or with a change in BMI (*n* = 478 samples), postmenopausal women not using hormone therapy (*n* = 577 of which 223 donated 2 samples), and control women (*n* = 940 of which 592 donated 2 samples). The stable BMI group includes participants with ≤2 kg/m^2^ change in BMI between the two blood collections. The groups with a change in BMI includes participants with >2 kg/m^2^ change in BMI between the two blood collections.

Participants	Median	Quartile 1–Quartile 3
All women	0.43	0.36–0.50
Fasting women	0.45	0.37–0.52
Women with stable BMI	0.43	0.36–0.51
Women with a change in BMI *	0.41	0.33–0.48
Postmenopausal women not using hormone therapy	0.44	0.36–0.53
Control women	0.44	0.37–0.51

* BMI change >2 kg/m^2^.

**Table 3:** Most and least stable metabolites. ICCs were estimated among four participant subgroups: all participants (*n* = 1184 repeated samples and *n* = 696 unique samples), fasting participants (*n* = 765 repeated and *n* = 544 unique samples), and among participants with stable (*n* = 706 repeated samples) or unstable BMI (*n* = 478 repeated samples). The stable BMI group includes participants with ≤2 kg/m^2^ difference in BMI between the two blood collections. The unstable BMI group includes participants with >2 kg/m^2^ difference in BMI between the two blood collections.

			Intra-Class Correlation			
	Metabolite Name	Metabolite Class	All	Fasting	Stable BMI	Unstable BMI
Most stable metabolites	N6,N6-dimethyllysine	Organic acids and derivatives	0.82	0.84	0.83	0.83
	Dimethylguanidino valerate	Other	0.72	0.73	0.73	0.71
	N-acetylornithine	Organic acids and derivatives	0.69	0.72	0.70	0.68
	C34:2 PC plasmalogen	Phosphatidylcholine plasmalogens	0.66	0.67	0.66	0.61
	C38:4 PC	Phosphatidylcholines	0.65	0.66	0.66	0.63
	Glycine	Amino acids	0.65	0.64	0.65	0.62
	C5-DC carnitine	Carnitines	0.64	0.63	0.66	0.62
	N4-acetylcytidine	Nucleosides, nucleotides, and analogues	0.64	0.62	0.68	0.62
	(A)Symmetric dimethylarginine	Organic acids and derivatives	0.62	0.63	0.66	0.65
	C36:1 PE plasmalogen	Phosphatidylethanolamine plasmalogens	0.62	0.62	0.64	0.59
Least stable metabolites	1-methylhistidine	Other	0.21	0.18	0.21	0.25
	4-hydroxyhippurate	Other	0.21	0.19	0.18	0.26
	Acetaminophen *	Other	0.2	0.18	0.21	0.21
	Guanosine	Other	0.2	0.20	0.17	0.23
	Allantoin	Other	0.18	0.21	0.15	0.20
	Hydroxyproline	Carboxylic acids and derivatives	0.18	0.12	0.17	0.17
	Methyl N-methylanthranilate	Other	0.17	0.13	0.14	0.22
	Trimethylamine-N-oxide	Other	0.16	0.15	0.12	0.24
	Ectoine	Other	0.09	0.08	0.08	0.10
	Palmitoylethanolamide	Other	0.05	0.03	0.05	0.06

* exogenous metabolite.

**Table 4:** Characteristics of study participants in the primary dataset.

	First Collection	Second Collection
*n*	1880	1184
Age, y	55.57 (6.92)	66.46 (6.87)
BMI, kg/m^2^	25.37 (4.53)	26.57 (5.11)
Physical activity, MET-hrs/wk	16.34 (20.00)	19.58 (20.78)
Alcohol consumption, g/day	6.71 (10.95)	5.81 (9.45)
AHEI ^	47.31 (10.67)	50.16 (9.98)
Menopausal status, %		
Premenopausal	479 (25.5)	8 (0.7)
Postmenopausal, no PMH ^#^ use	577 (30.7)	374 (31.6)
Postmenopausal, PMH ^#^ use	587 (31.2)	788 (66.6)
Missing/Dubious	237 (12.6)	14 (1.2)
Fasting (>8 h), %	1309 (69.6)	1062 (89.7)
Smoking, %		
Never	888 (47.4)	551 (46.7)
Past	748 (39.9)	575 (48.7)
Current	238 (12.7)	55 (4.7)
Race, %		
White	1853 (98.6)	1173 (99.1)
Black	14 (0.7)	4 (0.3)
Asian	10 (0.5)	5 (0.4)
Other/missing	3 (0.2)	2 (0.2)

^ Alternative Healthy Eating Index, calculated without alcohol consumption, ^#^ Postmenopausal Hormone.

## Data Availability

Data access must be approved by the institutional review boards of the Brigham and Women’s Hospital and Harvard T.H. Chan School of Public Health. Inquiries are encouraged through http://www.nurseshealthstudy.org/researchers.

## Supplementary Materials

The following supporting information can be downloaded at: https://www.mdpi.com/article/10.3390/metabo12050372/s1, Figure S1. Intra-class correlations for known metabolites and unknown metabolite features by participant strata, Table S1. Within-person stability over 10 years across different participant strata; Table S2. Intra-class correlations among unknown metabolite features in the primary dataset; Table S3. Intra-class correlations among unknown metabolite features across participant strata in the primary dataset; Table S4. ICCs for known metabolites measured in the primary and secondary dataset; Table S5. Characteristics of fasting participants in the primary dataset and all participants in the secondary dataset; Table S6. Reference standards and spectral evidence used to identify metabolites.
