# Supplementary material for: Intrapersonal Stability of Plasma Metabolomic Profiles over 10 Years among Women
Source: Metabolites. 2022 Apr 20;12(5):372. doi: 10.3390/metabo12050372 (PMC9147746; doi:10.3390/metabo12050372)
Supplement: Supplementary file 1 [file metabolites-12-00372-s001.zip › Supplementary_figure.zip!/Supplementary_figure.pdf]

Supplementary material

2.2. Metabolite profile stability over 10 years in the primary dataset among unknown metabolite features.

For unknown metabolite features, the median intra-class correlation (ICC) was 0.41 (Q1: 0.34; Q3: 0.48; Supplementary Table S2). ICCs among lipids and lipid-related unknown metabolite features were similar to ICCs among polar unknown metabolite features (median ICC: 0.41 vs 0.41, Wilcoxon rank sum test p = 0.07). ICCs differed significantly comparing metabolites with coefficient of variation (CV) < 25% with metabolites with CV ≥ 25% (median ICC: 0.43 vs 0.39, Wilcoxon rank sum test $p < 0.01$). The median % difference in unknown metabolite feature levels between the two collections, calculated based on raw values, was 0.03% (Q1: -6.80%, Q3:4.13%).

We observed statistically significant, albeit small, differences in unknown metabolite feature ICCs across participant strata in sensitivity analyses (Supplementary Table S3, and Supplementary Figure S1). Metabolite ICCs estimated among all women (median ICC = 0.41) were slightly different from ICCs estimated among fasting women (median ICC = 0.42; paired Wilcoxon test $p < 0.01$), from ICCs estimated among women with stable BMI (median ICC = 0.42; paired Wilcoxon test $p < 0.01$), and from ICCs estimated among women with a change in BMI (median ICC = 0.40; paired Wilcoxon test $p < 0.01$), and from ICCs estimated among control women (median ICC = 0.41; paired Wilcoxon test $p < 0.01$). ICCs estimated among all women were similar to ICCs estimated among postmenopausal women not using hormone therapy at either collection (median ICC = 0.41; paired Wilcoxon test p = 0.29).

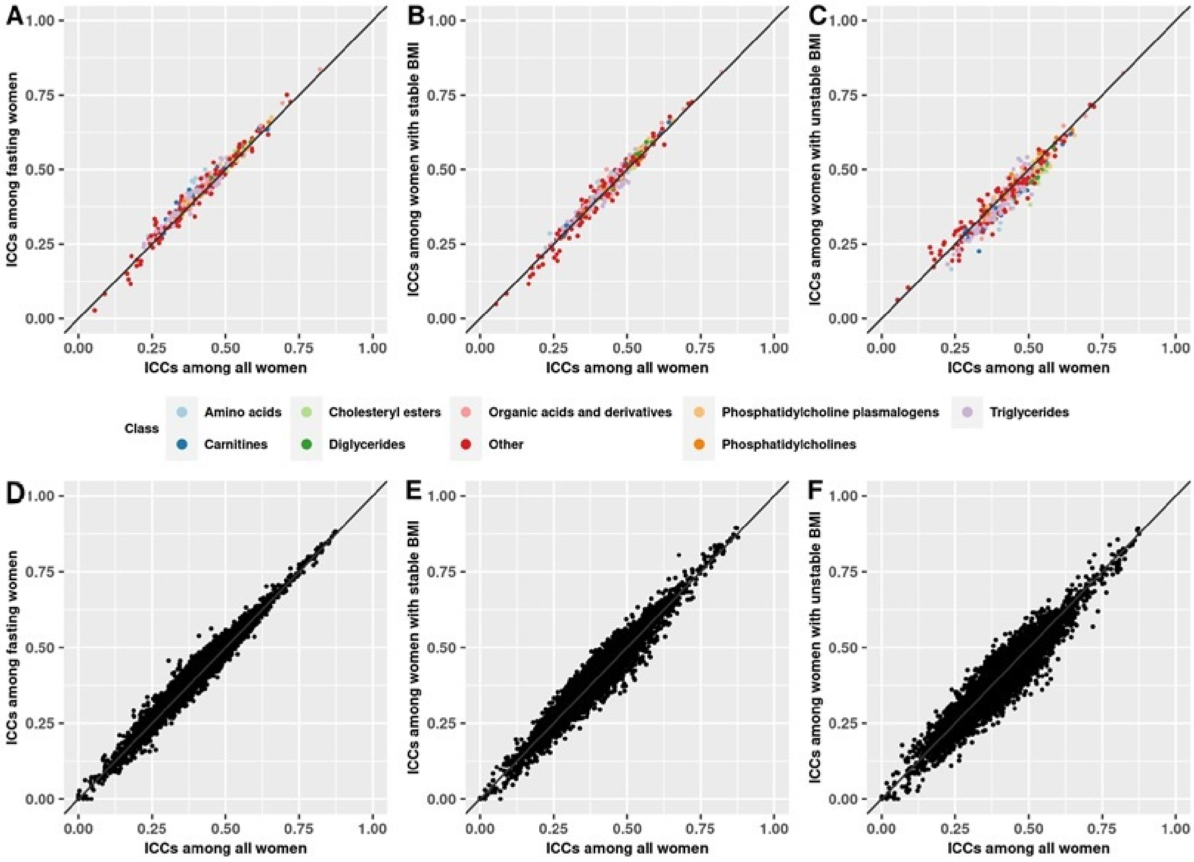

Supplementary Figure S1. Intra-class correlations for known metabolites and unknown metabolite features by participant strata. Intra-class correlations (ICCs) for known metabolites estimated among all women are plotted against ICCs estimated among fasting women (panel **A**), women with stable BMI (panel **B**) and women with unstable BMI (panel **C**). ICCs for

unknown metabolite features estimated among all women are plotted against ICCs estimated among fasting women (panel **D**), women with stable BMI (panel **E**) and women with unstable BMI (panel **F**). The stable BMI group includes participants with ≤2kg/m^2 difference in BMI between the two blood collections. The unstable BMI group includes participants with >2kg/m^2 difference in BMI between the two blood collections.
